# Advancing Mantle Cell Lymphoma Risk Assessment: Navigating a Moving Target

**DOI:** 10.1002/hon.70072

**Published:** 2025-06-15

**Authors:** Simone Ferrero, Simone Ragaini

**Affiliations:** ^1^ Hematology Division Department of Molecular Biotechnologies and Health Sciences University of Torino Torino Italy

**Keywords:** biomarkers, mantle cell lymphoma, minimal residual disease, personalized medicine, risk assessment, TP53

## Abstract

Mantle cell lymphoma (MCL) is a B‐cell malignancy characterized by t(11;14)(q13;q32) translocation and heterogeneous clinical behavior. Advances in risk stratification enabled the distinction of conventional MCL (cMCL) from non‐nodal MCL (nnMCL) subtypes, which presents with distinct biological and clinical features. Prognostic tools such as MIPI/MIPI‐c, Ki‐67 expression, and *TP53* mutation/deletion status have enhanced risk assessment, while new genomic alterations including 17p deletion, *CDKN2A* loss are emerging as a new prognostic factors. Above all, *TP53* disruption remains the major adverse factor, associated with poor outcomes despite new combination therapies. Although recent clinical trials are exploring innovative targeted strategies, the effective management of high‐risk MCL remains challenging till now. Minimal residual disease (MRD) monitoring is now emerging as a dynamic prognostic tool, offering potential for treatment adaptation. The integration of molecular and clinical predictors into personalized therapeutic strategies along with MRD monitoring could represent the basis of the future algorithm of MCL management.

## 1 Introduction

Mantle cell lymphoma (MCL) is a B‐cell malignancy defined by the characteristic t(11;14)(q13;q32) translocation and a highly heterogeneous clinical course. In recent years, increasingly accurate risk stratification of MCL has challenged conventional treatment algorithms, leading to a more personalized approach to therapy.

## 2 Not Only a Problem of Lymph‐Nodes: Nodal or Non‐Nodal MCL?

The variability in the clinical behavior of MCL is partly explained by the different biological characteristics of conventional MCL (cMCL) and non‐nodal MCL (nnMCL). Non‐nodal leukemic MCL (10%–20% of cases) is typically characterized by mutated immunoglobulin heavy chain variable region genes (IGHV), SOX11 negativity, and a leukemic presentation with splenomegaly. Despite the introduction of the L‐MCL16 assay and the nanostring‐based MCL35 proliferation signature to identify nnMCL [1, 2], robust criteria to distinguish nnMCL from cMCL are currently lacking.

## 3 MCL Risk at a First Glance

The clinical behavior of cMCL cases is highly variable. A comprehensive initial evaluation is essential to identify high‐risk disease (HRD) [3]. Cytomorphology helps to differentiate classical MCL from pleomorphic and blastoid variants, the latter being associated with an aggressive course [4, 5]. The Mantle Cell Lymphoma International Prognostic Index (MIPI), which includes age, performance status, serum lactate dehydrogenase and white blood cell count, stratifies patients into three risk groups with different outcomes [6]. Integration of MIPI with Ki‐67 expression ≥ 30% refines risk stratification (MIPI‐c), defining four risk groups with 5‐year overall survival (OS) rates ranging from 85% to 17% [7]. Notably, emerging evidence suggests that a Ki‐67 cut‐off of 50% may further improve MCL prognostication [8]. Finally, HRD is also identified by CNS involvement at diagnosis. Blastoid histology, B symptoms, elevated lactate dehydrogenase, Eastern Cooperative Group performance status ≥ 2 and a high Mantle Cell Lymphoma International Prognostic Index score appear to be associated with CNS involvement [9].

## 4 Molecular Refinement of High‐Risk Cases

A major biological risk factor in MCL is *TP53* disruption (mutation or 17p deletion), which leads to dysregulation of cell cycle control, apoptosis, DNA repair and senescence. TP53 aberrations correlate with aggressive disease features, including blastoid/pleomorphic histology, high Ki‐67 and complex karyotypes, and are associated with poor outcome [10]. *TP53* mutations have an overall prevalence of 10% in MCL patients [11]. The poor prognosis of *TP53*‐mutated MCL has been previously demonstrated in large patient cohorts from Nordic Lymphoma Group “MCL2/MCL3” [12] and FIL “MCL0208” [13] clinical trials. *TP53* mutated cases also continue to have a negative prognostic effect in patients who progress after 24 months from diagnosis, a subgroup known to be intrinsically characterized by a better prognosis than early POD patients [14]. Whether *TP53* mutation burden might play a role in MCL is still unclear. In the BOVen cohort (*N* = 25 patients) [15, 16], the variant allele frequency (VAF) of *TP53* SNV/indels detected by targeted sequencing was associated with poor PFS (HR 33.2; *p* = 0.05) but not OS (HR 28.6; *p* = 0.09). In contrast, in a comparator cohort of MCL patients treated with frontline standard chemo‐immunotherapy regimens [17], *TP53* VAF appeared to have the opposite effect, although this was not significant for PFS (HR 0.98; *p* = 0.2) or OS (HR 0.97; *p* = 0.06). Of note, few cases of *TP53* mutations in the peripheral blood of MCL patients can also be attributed to myeloid clonal hematopoiesis clones and their biological and prognostic role is currently unclear [18, 19]. In addition to *TP53* burden, the use of p53 protein expression as a surrogate marker for *TP53* mutation remains an open question. Indeed, immunohistochemical expression of p53 protein has previously been shown to correlate, at least in part, with *TP53* missense mutations [20, 21]. However, immunohistochemistry cannot identify all relevant mutations, has uncertain reproducibility, and should only be used when *TP53* DNA sequencing is not available. Interestingly, the combination of high p53 expression (> 50%) and high MIPI‐c has been shown to capture a group of MCL patients with worse PFS and OS in the MCL Younger [22] and MCL‐Elderly trials [23]. In addition to *TP53* mutations, deletions of *CDKN2A (p16)* and *TP53* were shown to have a poor prognostic value for both overall survival (OS) and time to failure (TTF) in the EMCL Younger cohort, independent of high‐dose cytarabine induction [24].

The adverse prognostic effect of *CDKN2A/B* deletions, as well as the additive adverse prognostic effect in combination with *TP53* deletions, was also confirmed in the international, randomized, phase III MCL Younger and MCL Elderly trials of the European MCL Network and in the Fondazione Italiana Linfomi V‐RBAC trial [20, 25]. Similarly, genomic complexity, as measured by the number of CNAs, correlates with poorer OS [20, 26]. In this context, a complex karyotype, defined as the presence of ≥ 3 unrelated chromosomal abnormalities, not including t(11;14). was associated with a shorter median OS after chemotherapy than those with a non‐CK (4.5 vs. 11.6 years; *p* < 0.01) [27]. Finally, *MYC* amplification or translocation is associated with poor prognosis [28] and interestingly, *TP53* and *MYC* aberrations appear to add negative prognostic value independent of genomic complexity [26].

The increasing role of the genetic landscape of MCL has led to the implementation of conventional prognostic indices such as MIPI and MIPIc with molecular features to further refine risk. In this context, a MIPI‐genetic score (MIPI score combined with *KMT2D* mutation and *TP53* aberration status) was able to identify a high‐risk group with 4‐year PFS and OS rates of 11% and 45%, respectively. Subsequently, the addition of 4 CNAs to the MIPIg allowed better risk stratification in the cohort of the FIL MCL0208 trial (MIPI‐go) [29]. Although the novel molecular scores need to be validated in larger patient cohorts, they represent the new frontier of risk classification.

## 5 The Unmet Need of Treating High‐Risk MCL

Effective treatment of *TP53‐*mutant MCL remains an unmet need. Recent evidence from the TRIANGLE trial suggests that ASCT may still have a role in p53‐expressing MCL after the addition of ibrutinib to standard chemoimmunotherapy. In fact, the ASCT + ibrutinib arm improved patients' failure‐free survival (HR 0.14 [98.3% CI 0–0.57]) compared with standard therapy [30]. In the SHINE trial [31], elderly patients did not have a PFS benefit with the addition of ibrutinib to R‐bendamustine regimen in high‐risk MIPI (HR 1.0; 95% CI 0.7–1.5) or mutated *TP53* (HR 0.9; 95% CI 0.5–1.8) and blastoid histology (HR 0.7; 95% CI 0.3–1.3). In ENRICH trial [32], comparing Ibrutinb‐R with R‐chemotherapy, both with rituximab maintenance in patients ≥ 60 years with untreated MCL, no significant PFS benefit was observed in *TP53* mutated and blastoid patients. Similarly, in the ALTAMIRA trial [33], patients received MRD‐guided treatment with 6 cycles of acalabrutinib plus rituximab followed by maintenance for a total of 3 years. High‐risk patients (*TP53* mutated with blastoid histology) were treated until progression, while low‐risk patients discontinued acalabrutinib if MRD negative after a minimum of 1 year of treatment. Notably, high‐risk patients, defined by the addition of ki67 ≥ 30% to *TP53* mutation and blastoid histology, showed a median 2‐year PFS of 38% compared to 96% in the low‐risk group. In the SYMPATICO study, in patients with relapsed/refractory MCL, the combination of ibrutinib/venetoclax approximately doubled median PFS compared to ibrutinib plus placebo in both the *TP53‐*mutated subgroup (20.9 vs. 10.9 months) and the *TP53* wild‐type subgroup (46.9 vs. 22.2 months) [34].

The triplet combination of zanubrutinib + obinutuzumab + venetoclax in the BOVen study with minimal residual disease (MRD)‐guided discontinuation after 24 cycles of therapy showed a 2‐year PFS of 72%, improving the results of historical cohorts. In the VIPOR trial [35], a fixed‐duration, multi‐agent targeted regimen (ViPOR) of 6 cycles of venetoclax, ibrutinib, obinutuzumab and lenalidomide without maintenance or consolidation was tested in 20 newly diagnosed and 16 relapsed MCL pts. At 2 years, 100% of *TP53* mutated/deleted, 81% of Ki‐67 > 30%, 80% of post‐BTKi and 74% of blastoid patients remained free of progression.

Sub‐analyses at 3‐year follow‐up of the ZUMA‐2 trial [36] in a limited subset of cases did not show the expected differences in complete remission rates and median PFS between *TP53* mutant and *TP53* wild‐type cases (CRs, 100% and 70%, respectively; mPFS, not achieved, vs. 47. 6 months), classic and blastoid histology (CRs 65% and 53%, respectively; mPFS, 18.2 [7.8–47.6] vs. 14.5 months [3.0–48.0]) and Ki67 index < 30% or ≥ 30% (CRs 78% vs. 72%, respectively; mPFS, 27.5 months vs. 46.6). On the other hand, in the large, “real‐world evidence” retrospective study by the US Lymphoma CAR T Consortium [37], PFS was inferior in patients with high‐risk MIPI (HR 3.82; *p* < 0.001), Ki‐67% ≥ 50% (HR 3.02; *p* = 0.007), *TP53* mutation or deletion (HR 1.98; *p* = 0.008), complex karyotype (HR 2.23; *p* = 0.005) and blastoid/pleomorphic variant (HR 1.61; *p* = 0.036). In the Phase 2 TARMAC study [38] 21 R/R MCL patients after ≥ 1 prior line of therapy, (which could include a BTKi) received Ibrutinib commenced before leukapheresis and continued through CAR‐T manufacture for a minimum of 6 months after CAR‐T administration. Among patients with *TP53*‐mutation data available, mutated (*n* = 8) and WT patients (*n* = 10) showed similar PFS after 13‐month median follow‐up. Finally, results from a limited subset of the BRUIN study showed that pirtobrutinib, a non‐covalent BTKi, had ORRs that were slightly influenced by blastoid histology, *TP53* mutation and Ki‐67 ≥ 30% (50%, 47%, and 56%, respectively). Table 1 summarizes outcomes of *TP53*‐mutated vs *TP53* wild type MCL patients treated with standard chemo‐immunotherapy approach or innovative target therapy combination.

**TABLE 1:** Clinical trials based including standard chemo‐immunotherapy and/or innovative target drugs in treatment‐naïve and relapsed/refractory mantle cell lymphoma.

Trial	Patients enrolled^a^	Regimen	*TP53*‐mutated MCL	*TP53* wild‐type mutated MCL
MCL2 and MCL3 [12]	Treatment‐naïve	Induction: Alternating R‐CHOP/R‐high‐dose cytarabine → consolidation: High‐dose chemotherapy and ASCT	PFS HR 6.8 (95% CI 3.4–13.8)	—
	*N* = 176			
FIL MCL0208 [13]	Treatment‐naïve	3 R‐CHOP‐21 + R‐high‐dose cyclophosphamide + R‐high‐dose Ara‐C → ASCT → randomization to lenalidomide	PFS HR 3.4 (95% CI 2.1–5.4) in *TP53* disrupted (mutation/deletion) patients	—
	*N* = 186			
SHINE [31]	Treatment‐naïve	Ibrutinib + BR versus placebo + BR	Ibrutinib + BR: mPFS: 28.8 months	Ibrutinib + BR: mPFS: 80.6 months
	*N*: 140 in ibrutinib + BR group			
FIL V‐RBAC [25]	Treatment‐naïve	R‐BAC + venetoclax in high risk MCL versus R‐BAC in low risk MCL	3‐year PFS 30.8%	3‐year PFS 79.9%
	*N*: 132			
TRIANGLE [30]	Treatment‐naïve	R‐CHOP/R‐DHAP → ASCT → R maintenance versus ibrutinib + R‐CHOP/R‐DHAP → ASCT → ibrutinib/R maintenance	High p53 IHC expression (> 50%): MIPI‐adjusted HR: 0.68 (95% CI 0–1.80) favoring A + I versus A	Low p53 IHC expression (< 50%): MIPI‐adjusted HR: 0.85 (95% CI 0–1.44) favoring A + I versus A
	*N*: 358			
BOVen [15]	Treatment‐naïve	Zanubrutinib + obinutuzumab + venetoclax	2‐year PFS: 72%	—
	*N* = 25			
ALTAMIRA [33]	Treatment‐naïve	Acalabrutinib + rituximab	High risk (ki67 ≥ 30%/*TP53* mutation/blastoid histology): 2 years‐PFS 38%	Low risk (ki67 < 30%/*TP53* wild‐type/no blastoid histology): 2 years‐PFS 96%
	*N* = 81			
SYMPATICO [34]	Treatment‐naïve and relapsed/refractory	Ibrutinib + placebo versus ibrutinib + venetoclax	Ibrutinib + Ven: mPFS 20.9 months (95% CI 14.7–30.6)	Ibrutinib + Ven: mPFS NR (95% CI 36.4–NE)
	*N*: 193			
VIPOR [35]	Treatment‐naïve and relapsed/refractory	Venetoclax + ibrutinib + obinutuzumab + lenalidomide	2‐year TTP 100%	
	*N* = 36			
VALERIA [39]	Relapsed/refractory	Venetoclax + lenalidomide + rituximab	12‐month OS 67% (95% CI 48–92), PFS 33% (95% CI 17–64)	12‐month OS 86% (95% CI 74–100), PFS was 71% (95% CI 57–90)
	*N* = 46			
BRUIN [40]	Relapsed/refractory	Pirtobrutinib	ORR 47% (95% CI 33%–80%)	ORR 58% (95% CI 23%–72%)
	*N* = 36		mDOR 17.6 (95% CI 1.7–NE)	mDOR 14.8 (95% CI 1.9–NE)

Abbreviations: ASCT, autologous stem cell transplantation; BR, bendamustine‐rituximab; CI, confidence interval; HR, hazard ratio; IHC, immunohistochemistry; mDOR, median duration of response; NR, not reached; ORR, overall response rate; OS, overall survival; PFS, progression‐free survival.

^a^ Data referred to the *TP53*‐analyzed patients.

Future perspectives in high‐risk MCL include the attempt to move CAR‐T cells therapy in first line. In particular, the ongoing CARMAN study [41] including brexucabtagene autoleucel following a shortened induction with chemo‐immunotherapy plus BTKi compared to standard of care, is enrolling MCL patients with MIPI‐c high‐intermediate or high risk and/or *TP53* mutation and/or p53 overexpression by immunohistochemistry.

## 6 Moving From Baseline to Dynamic Prediction of MCL Risk: The Role of Longitudinal MRD Assessment

Minimal residual disease (MRD) has gained considerable interest following the publication of several reports demonstrating its high predictive value in this subtype of lymphoma. The detection of MRD is currently based on the standardized real‐time quantitative (RQ)‐PCR method according to the EuroMRD guidelines, with a median sensitivity of up to 1 × 10^−5^, allowing the identification of a marker such as a BCL1 or IGH translocation in approximately 80% of patients. Recent efforts have focused on next‐generation sequencing (NGS)‐based approaches, both immunoglobulin‐based and capture‐based, performed on peripheral blood (PB) or bone marrow (BM) samples [42, 43]. The role of MRD in MCL has been the focus of the two large international Phase 3 trials MCL Younger [22] and MCL Elderly [44], which evaluated respectively the role of different induction protocols followed by either 2 different high‐dose regimens with ASCT (MCL Younger) and 2 different maintenance regimens (MCL Elderly). In this setting, patients who achieved molecular remission after induction showed a significantly improved response duration compared to patients with residual disease [45]. The effect of MRD positivity on time to progression over time was subsequently demonstrated in the FIL MCL0208 trial [46], where the Authors also proposed a time‐varying kinetic model based on the combination of regularly updated MRD results and MIPI as the best way to provide a powerful risk stratification tool suitable for MRD‐guided treatment, rather than the information provided by a single “punctual” MRD time point. The role of MRD in MCL is particularly important at the end of induction therapy to determine who will benefit from maintenance rituximab. This was investigated by Hoster et al. in a subset of patients from the European Mantle Cell Lymphoma Elderly Trial, designed to test how MRD status influenced the efficacy and impact of maintenance therapy [47]. The results showed that response duration was prolonged with rituximab maintenance in patients who were MRD negative at the end of induction (hazard ratio [HR] = 0.38; 95% CI 0.21–0.63). In addition, in the same series, OS was prolonged with rituximab maintenance in patients who were MRD negative (HR = 0.37; 95% CI 0.20–0.68). Conversely, in the same study, the efficacy of rituximab maintenance appeared reduced in patients scoring MRD‐positive after induction therapy; these results is in line with findings from lenalidomide maintenance in FIL MCL0208 trial, too [46]. Strategies to treat MRD‐positive patients more effectively are being explored. For example, the use of rituximab plus lenalidomide as maintenance proposed in ELDERLY R2 trial [48] did not improve the outcome of MRD+ patients after induction therapy. Evidence from the TRIANGLE trial data embedded in the Multiply Project will investigate whether ibrutinib maintenance might have a role in MRD+ patients after induction‐therapy.

## 7 How I Treat MCL (If I Could)

**FIGURE 1:**
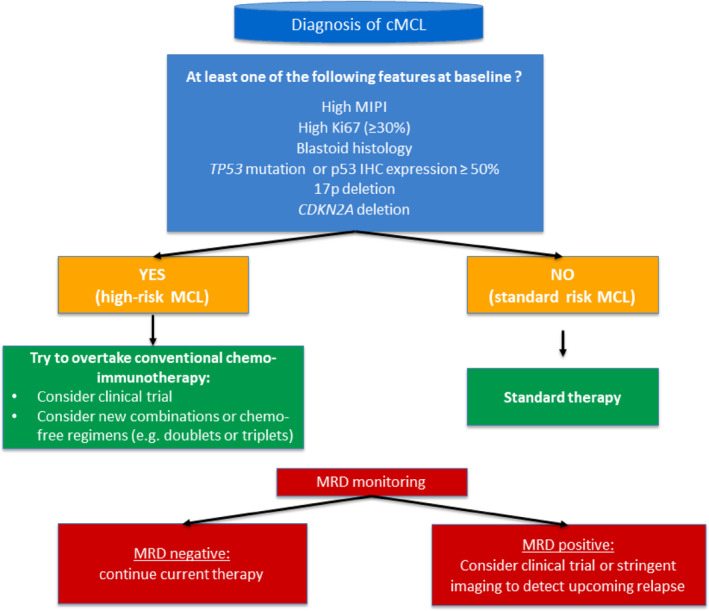
Proposed risk assessment and potential clinical management of MCL patients.

The advancing in MCL risk assessment is becoming crucial in everyday clinical decision making. While a better deciphering of genomic complexity of MCL is needed to further refine high‐risk patients, an adequate treatment strategy should be found for high‐risk disease. Based on current evidence, the presence of at least one of the following factors should lead clinicians to consider MCL patient as high‐risk: (1) High MIPI; (2) High Ki67 (≥ 30%); (3) Blastoid histology; (4) *TP53* mutation (or p53 IHC expression ≥ 50% if mutation data is not available); (5) 17p deletion; and (6) *CDKN2A* deletion. Low‐risk patients should be treated with standard‐of‐care therapy according to age and frailty status. High‐risk patients should be enrolled in a clinical trial and if not available should be treated with new combinations [TRIANGLE] or chemo‐free regimens including target‐therapy‐containing doublets or triplets [BOVEN, VIPOR, SYMPATICO]. MRD monitoring should be performed during and after induction therapy. MRD‐negative patients should continue maintenance therapy while MRD‐positive cases should be enrolled in dedicated clinical trials (Figure 1).

## 8 Conclusions

Risk assessment in MCL remains an evolving challenge, with limitations primarily related to routine clinical applicability. While a combined approach integrating clinical, pathological and genomic factors may improve risk stratification and therapeutic decision‐making, dynamic MRD assessment tools offer promising prospects but require validation and integration into clinical practice. However, for the first time in a long time, the ongoing refinement of MCL risk models is likely to bring us a step closer to capturing the true biological complexity of the disease.

## Conflicts of Interest

S.F. is a consultant for Janssen, EUSA Pharma, Abbvie and Sandoz; is on the advisory board of Janssen, EUSA Pharma, Recordati, Incyte, Roche, Astra Zeneca and Italfarmaco; received speaker’s honoraria from Janssen, EUSA Pharma, Recordati, Lilly, Beigene, Gilead and Gentili; and received research funding from Gilead, Beigene and Morphosys. S.R. received speaker’s honoraria from Beigene.

## Peer Review

The peer review history for this article is available at https://www.webofscience.com/api/gateway/wos/peer-review/10.1002/hon.70072.

## Permission to Reproduce Material From Other Sources

The authors have nothing to report.

## Data Availability

The authors have nothing to report.
